# From microarray to biology: an integrated experimental, statistical and in silico analysis of how the extracellular matrix modulates the phenotype of cancer cells

**DOI:** 10.1186/1471-2105-9-S9-S4

**Published:** 2008-08-12

**Authors:** Mikhail G Dozmorov, Kimberly D Kyker, Paul J Hauser, Ricardo Saban, David D Buethe, Igor Dozmorov, Michael B Centola, Daniel J Culkin, Robert E Hurst

**Affiliations:** 1 Department of Urology, Oklahoma University Health Sciences Centre, Oklahoma City, OK 73104, USA; 2 Department of Biochemistry and Molecular Biology, Oklahoma University Health Sciences Centre, Oklahoma City, OK 73104, USA; 3 Microarray Core Facility, Oklahoma Medical Research Foundation, Oklahoma City, OK 73104, USA; 4 Department of Physiology, Oklahoma University Health Sciences Centre, Oklahoma City, OK 73104, USA

## Abstract

A statistically robust and biologically-based approach for analysis of microarray data is described that integrates independent biological knowledge and data with a global F-test for finding genes of interest that minimizes the need for replicates when used for hypothesis generation. First, each microarray is normalized to its noise level around zero. The microarray dataset is then globally adjusted by robust linear regression. Second, genes of interest that capture significant responses to experimental conditions are selected by finding those that express significantly higher variance than those expressing only technical variability. Clustering expression data and identifying expression-independent properties of genes of interest including upstream transcriptional regulatory elements (TREs), ontologies and networks or pathways organizes the data into a biologically meaningful system. We demonstrate that when the number of genes of interest is inconveniently large, identifying a subset of "beacon genes" representing the largest changes will identify pathways or networks altered by biological manipulation. The entire dataset is then used to complete the picture outlined by the "beacon genes." This allow construction of a structured model of a system that can generate biologically testable hypotheses. We illustrate this approach by comparing cells cultured on plastic or an extracellular matrix which organizes a dataset of over 2,000 genes of interest from a genome wide scan of transcription. The resulting model was confirmed by comparing the predicted pattern of TREs with experimental determination of active transcription factors.

## Background

Microarrays are widely used to overview gene expression landscapes under different experimental conditions. Since their initial appearance microarrays developed into very dependable tools with good inter- and intra-platform reproducibility [1,2]. Although numerous attempts to unify microarray analysis workflow were made, each manufacturer has its own methods for processing large quantities of data, and there is no general consensus as to the best means to analyze microarray data, and probably never will be. Each experimental situation is different, and different designs may be necessary for hypothesis testing as compared to hypothesis generation. With the former, one biological system is compared with another, and the significance of differences is statistically tested using a t-test, generally with the assumption that each biological sample is homogeneous. In such experiments statistical power becomes the driving consideration. In hypotheses generating experiments, a number of biological situations are compared, for example a series of different cell lines, a time course study or a dose-response study. The biological samples may not be homogeneous. Cost becomes a major consideration because the number of replicates needed to test hypotheses may make experiments prohibitive. Thus, there is a need for analytical approaches to use under hypothesis-generating conditions that are based on sound statistical principles but which nonetheless reduce the number of replicates needed to assemble at least a preliminary global picture of the effect of a particular biological situation on gene expression [3].

We present here a statistically robust approach for analyzing the changes in the transcriptome that is driven by the underlying biology. Previous work by I. Dozmorov showed that approaches based on separating variability in expression of genes into biological and technical sources provide an alternative means of identifying "genes of interest" for further analysis [4-7]. Under the assumption that in any experiment most genes do not change expression, the F-test is used to identify genes that express more variability than the overall technical variability of the system. This set of genes is referred to as "hypervariable genes," and has been assumed to reflect the relevant biological variables in the system. In this communication we have added a number of in silico tests based upon properties of these genes that do not depend upon expression. These additional analyses confirmed that at the level of transcriptional regulatory networks this approach does identify important genes that can then be assembled into networks of functions, transcriptional regulation and with previous knowledge. This represents a further extension of work published in our laboratory that included only *in silico *analyses [8,9].

We applied this method to examining the effect of cancer remodeled extracellular matrix (crECM) on bladder papilloma-derived cell line (RT4) as they grow over the course of several days on a crECM after having been transferred from culture on plastic [10]. Papillomas represent a very early premalignant change, and determining how a crECM can drive them toward malignancy could identify novel targets for therapy. Genes exhibiting major changes in expression introduced by crECM were selected and their functions examined. Two overlapping canonical pathways were identified as the main targets. Finally, transcription factors regulating the genes of interest were found and their validity proven by additional experimental method. Such integrative approach may reveal new roles of unknown genes [11], new drug targets [3], and lead to clinical tests [12].

## Results

**Figure 1:**
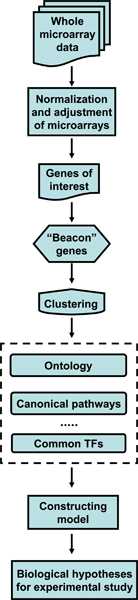
Schematic diagram of steps in microarray analysis.

**Figure 2:**
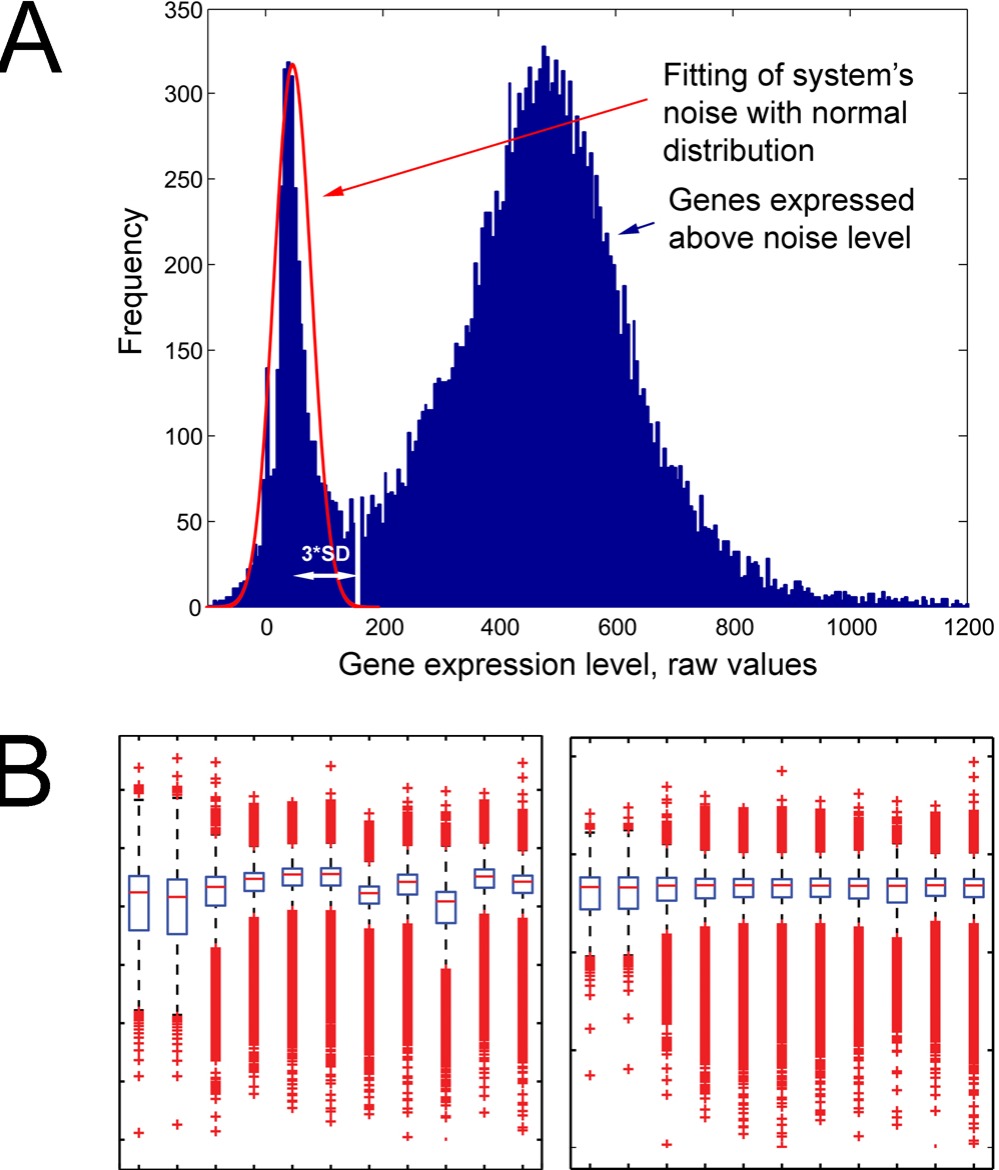
**Identification of noise level and microarray adjustment**. A) Frequency histogram of gene expression level from one microarray dataset. The first peak in the bimodal distribution represents the normal distribution of system noise centered around zero. Genes expressed 3 SD above noise level are defined as expressed genes. B) Box plots of microarray datasets before and after linear regression, values are log10 transformed.

The workflow chart of our approach is shown in Figure 1. The normalization process is presented because it is different from most approaches using normalization to the mean, median, or housekeeping genes. The frequency histogram of the un-normalized expression values yielded a bimodal, right-skewed curve as shown on Figure 2A[9]. The distribution around the peak near zero was fitted to a Gaussian curve, providing a measure of the variability around zero that can be used to identify genes expressed significantly above zero. The zero point itself is slightly above zero because of non-specific binding. Interestingly, the array contained completely blank spots, which show up as a sharp peak exactly around zero, as well as a large number of spots of the solvent (3× SSC) the long oligo clones are contained in. The average of those points corresponds almost exactly to the zero point established from the entire array by the method above. The standard deviation of this peak is used to normalize all the expression data. Normalizing expression to the uncertainty in zero allows for a ready determination for the threshold of non-zero expression. Thus, expression value of "3" corresponds to 3 standard deviations (SD) above the zero point and corresponds to the valley in the total distribution. With this value, the p-value of a false positive assignment of expression vs. non-expression is < 0.001. With a threshold of 5 SD, the p-value for a false positive is p < 2.87 × 10^-7^. The arrays were then Log10 transformed and globally adjusted to each other by robust linear regression, which assumes that the expression of most genes is not altered in the experiment and down-weights the effect on global expression of those that do change. Box plots (Figure 2B) graphically show this adjustment. Full sets of raw and transformed data are available on Gene Expression Omnibus (GEO, ), accession number GSE9291.

### Gene expression dynamics

**Figure 3:**
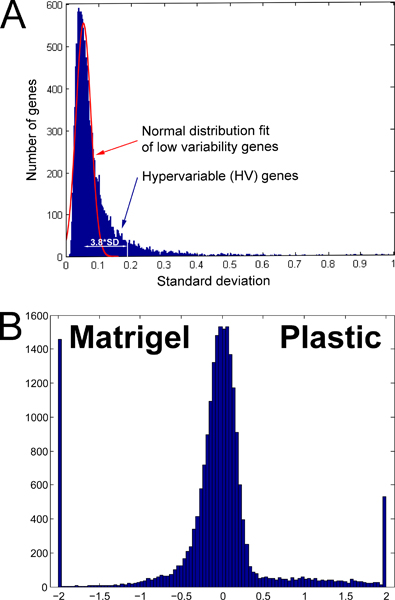
**Identification of hypervariable and differentially expressed genes**. A) Frequency histogram of variances of genes across timecourse. The normal distribution of low-variable genes identified from the left part of the histogram; the white vertical line marks the threshold for hypervariable genes expressed 3.8 SD above distribution of constant genes expressing only technical variability. B) Log10 ratio of average gene expression of cells grown on plastic and crECM is presented as a frequency histogram. Ratio values > 2 or < -2 were truncated and set to 2 and -2, respectively.

Genes expressed 5 SD above background were considered highly significantly expressed. While this choice is arbitrary, 5 SD above background was selected to focus on highly expressed genes and to minimize false positives by eliminating the noisy low-expressed genes. Of the 21,308 unique probes represented on the array, a total of 15,287 were expressed at 5 SD above background on the two arrays from cells grown on plastic and on the nine arrays from cells grown on crECM. The next step is to identify genes whose expression level responds to crECM. The total variance in expression of any gene is the sum of the technical variance (variance due to the measurement itself), Vt, the relevant biology, Vr, and the irrelevant biology Vi. Vi may be attributed to deviations in preparation of biological samples or other biological factors not related to the biology of interest. Genes that do not respond to the biological variables will express only technical variability and can effectively serve as "housekeeping genes" or a reference set against which genes that do respond can be identified with an F-test. Genes responsive to the biology generally show high variability that likely is systematic across the experiment. The overall mean technical variability was determined from the mode of the frequency distribution histogram of standard deviation of the data set (Figure 3A) and was 8.41% with a standard deviation of 6.75%. Thus, genes with relative standard deviations exceeding 3 SD above the mean (28.56%) were defined as hypervariable. A total of 3462 hypervariable (HV) genes were identified. However, the lack of replicates leads to an excessive sensitivity to single outliers. A second screening by a leave-one-out method [13] minimized false positives due to a single data point. A total of 2743 HV genes were judged to be hypervariable (p < 6.7 × 10^-5^) and, presumably, represent all the genes that respond to the presence of a crECM. This approach has proven reliable in identifying a set of genes of interest with minimal need for replicates as compared to methods based on t-tests [4,9].

### Visualization of gene expression changes

**Figure 4:**
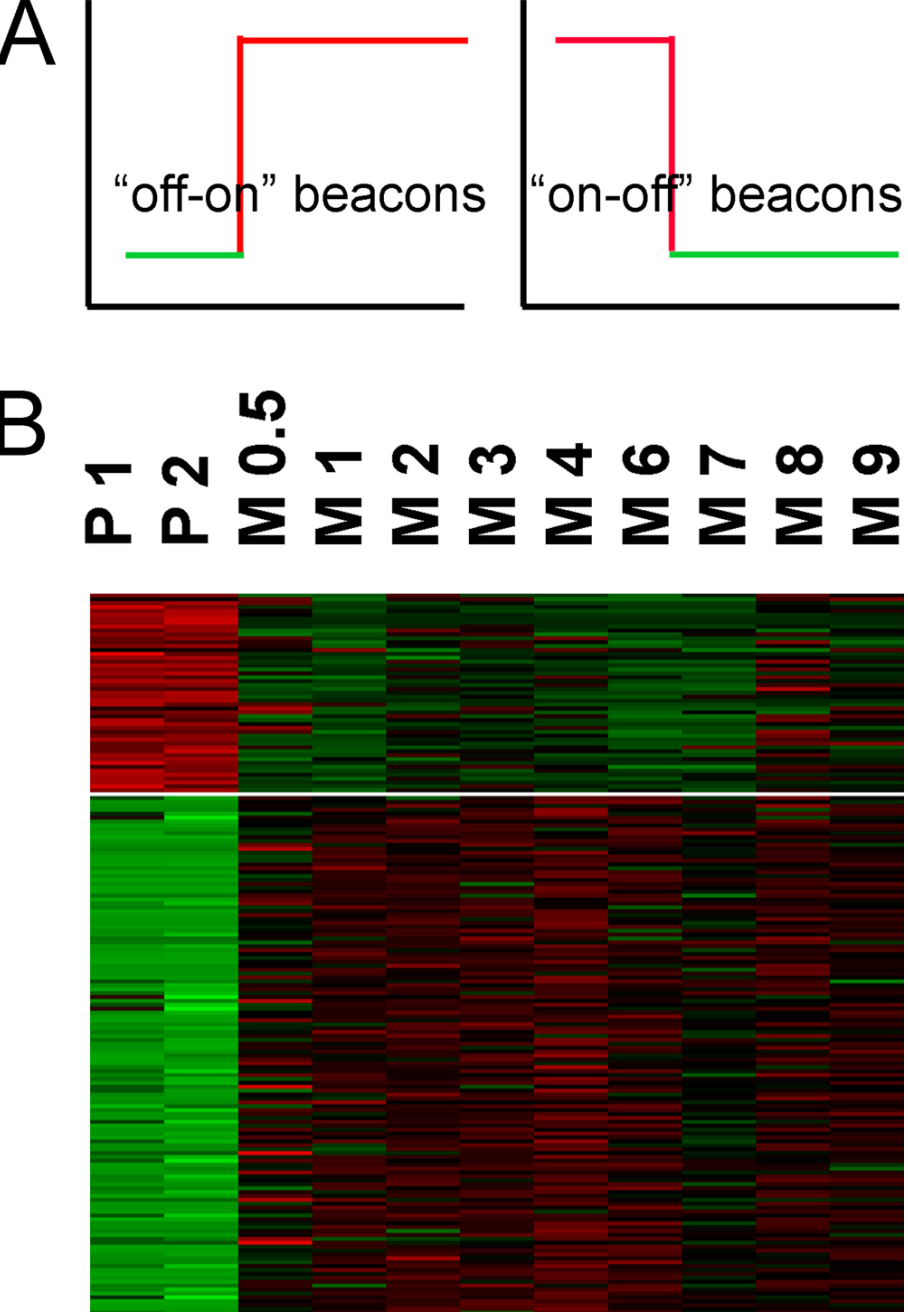
**Visualization of changes in gene expression level identified by microarray analysis**. A) Schematic graphs of main changes observed in the system from clustering of hypervariable genes. B) Part of clustering heatmap of "beacon" state change genes: P 1, P 2 – duplicate gene expression profiles of RT4 cells grown on plastic; M 0.5, M 1, M 2 etc. – cells grown on crECM for the indicated number of days. Red/green intensity indicates level of gene expression, up-/downregulated, respectively.

For easy visualization of up- and downregulated genes their expression values were antilog-transformed and normalized around zero with standard deviation equal to 1. They were organized into 3 clusters by K-means clustering with 1000 runs and a similarity metric as correlation (uncentered). Increasing number of clusters yielded similar clusters differing only in amplitude, while using 2 clusters failed to distinguish the obvious dynamics. The results were visualized with Java TreeView program [14,15] (data not shown). The clustering of hypervariable genes demonstrated that the vast majority of the biological variability was between cells grown on plastic and on the crECM, and with the main pattern being a change in state of expression, i.e. from off to on or vice versa. After the first 12 hours on the crECM, little variability in gene expression was observed. This indicates the changes of gene expression level occurred within the first 12 hours in cells grown on crECM, which in turn drives phenotypic changes (Figure 4). That later time points are not different from the earlier ones indicates no new biological processes such as cell death were introduced over the time course of the experiment.

This represents a very large set of genes of interest, and other than determining ontologies, such a large number is inconvenient to interpret. We therefore focused on the genes showing the largest changes, considering they could serve as "beacons" to draw our attention to the changes in underlying processes. Because the dynamics of gene expression exhibit mainly a change in state between cells growing on plastic vs. crECM, the set of hypervariable genes was filtered to identify the genes that are expressed below noise level on plastic and highly expressed on crECM ("off-on" genes) and vice versa ("on-off" genes) (Figure 4A). The two arrays of gene expression on plastic and nine arrays of timecourse on crECM were each averaged and genes with average expression level on plastic < 0 and on crECM > 1 (Log10 scale, average identifies geometric mean) were selected as "off-on" genes. The opposite criteria were applied to identify "on-off" genes. Using these stringent criteria, a total of 877 unique "off-on" genes were turned on by the crECM, whereas a total of 74 unique "on-off" genes were shut down by the crECM. The validity of this approach was tested in the next section.

Beside the main dynamic of state change genes a smaller number of genes showed change in level. Figure 3B shows the distribution of the ratio of expression of genes from cells grown on plastic to those grown on crECM. The standard deviation of this ratio is 0.2, and 3 standard deviations (0.6) corresponds to a 3-fold difference. A total of 241 genes were identified that were expressed at least 5 SD above background in cells grown on plastic and showed at least a 3-fold increase in expression. Only 67 genes showed the opposite pattern being highly expressed on plastic and decreasing 3-fold but still expressed above noise.

### Gene ontology analysis and visualization

The ontologies of the genes of interest were examined using the Database for Annotation, Visualization and Integrated Discovery (DAVID, ) [16] tool, which examines all the functions represented by each gene in a gene list and identifies groups that share ontologies. The over-represented ontologies form the basis for identifying functional processes represented in the change of state induced by a crECM. Several parameters can be adjusted to achieve a reasonable and comprehensive set of ontologies and associated genes. For the 877 "off-on" genes the following parameters were set: Similarity term overlap: 5; Similarity threshold: 0.5; Initial group membership: 5; Final group membership: 5; Multiple linkage threshold: 0.5, which is equal to the "Highest" stringency setting in DAVID. After examining the results provided by different stringencies, the above set was selected because the picture presented overall affinities without too many groups but provided sufficient detail to build a conceptual model of the effect of crECM on progressing urothelial cells.

Out of the 877 "off-on" genes 190 clustered into 12 clusters of ontologies at highest stringency and 86 did not have recognized ontologies. These 86 unannotated genes likely represent either novel processes not currently identified or genes whose participation in known processes has not yet been discovered [11]. The remaining 601 were predominantly distributed among "related genes" that shared some ontological features with one or more of the 12 clusters but did not rise above the threshold of significance. Some were entirely irrelevant and showed no similarity to any of the clusters. This step is illustrated in Table 1 along with significantly over-represented TREs shared by all members of each cluster.

**Table 1:** Major functional groups overrepresented among state-change genes and corresponding overrepresented TREs. The groups are presented according to the order of significance identified by DAVID. Overrepresented TREs marked in bold are either "off-on" TFs or increased their level; regular – not present in Panomics set; *italics *– not present under either condition.

**Functional group**	**Number of genes**	**Major Gene Ontologies**	**Overrepresented TREs**
*Gene expression, RNA processing and protein synthesis*			
12	73	Transcription factors	
7	5	Translation initiation factors	**v-Maf**, SOX-9, FOXJ2, CP2, **HFH-3**, **Elk-1**, NRF-2, *AREB6*
9	6	RNA processing – ribosome biogenesis	NGFI-C, GR, *HNF-4*, **YY1**, **Elk-1**, NRF-2, **v-Myb**, **NF-κB**, TATA, *c-Myc*
11	8	Zinc binding	MIF-1, **Tax/CREB**
*Cell signaling proteins*			
3	16	G-protein receptor	
2	7	GABA receptor/ion channels	N-Myc
6	9	Ion channels, K	
*Post-translational modification and regulatory control*			
8	5	Glycosyltransferases	
10	28	Kinases	**CDP**, CR3+HD, *CRE-BP1*, **CCAAT**
*Cell-ECM adhesion*			
4	4	Cadherins	**HNF-3β**, **CDP **CR3+HD, **E2**, **NF-κB**, **USF**
*Immune function associated with suppression of effector T-cells*			
1	15	Transmembrane immunoglobulin-like proteins	**NF-κB**, **v-Maf**, **RSRFC4**, FOXJ2, **AP-1**, **Pax-4**, **USF**, **CDP**, Brn-2
*Transmembrane proteins of unknown significance*			
5	14	Transmembrane proteins	**AP-1**

Examination of 241 level-change genes increased on crECM by medium stringency ontological analysis of these genes found five clusters of functions, three of which were similar to those for state change.

The 74 genes that were shut off and the 64 genes that were 3-fold down-regulated on crECM were less informative than were those that were turned on or up-regulated. At same stringency as was used for "off-on" genes, one over-represented cluster was identified in the genes that were shut off and consisted of 6 transcription factors sharing homeobox ontology. Decreasing the stringency to "medium" (the default for DAVID) increased the number of genes in the cluster to 10 but did not add clusters. Genes that were down-regulated at least 3-fold yielded two clusters under medium stringency.

The validity of the selection of "beacon genes" was tested by comparing the ontological clusters observed with the entire set of 2743 HV genes. A total of 17 clusters was seen, all of which were identified using the "beacon" genes. This demonstrates that the smaller data set of state- and level-change genes will identify all the processes seen in the larger set of HV genes.

### Pathway analysis

Having preliminary understanding of functions represented by state change genes, they were probed for membership in canonical pathways by Ingenuity^© ^Pathway Analysis (IPA, ). IPA maps each gene identifier to its corresponding gene object in the Ingenuity^© ^Pathways Knowledge Base, and generates multiple biological networks with associated ontologies from a list of focus genes, as well as general gene ontologies overrepresented. IPA canonical pathways analysis identified the most significant known biological pathways for a given set of genes. For identification of a significant canonical pathway or pre-defined network of genes it is only necessary to identify a single member as significant, hence the term "beacon" genes. The participation of other members of the network is checked manually against the entire data set to ensure they are expressed, or show a smaller change than the "beacon" genes threshold [9]. Pathways or connections involving genes that are not expressed are deleted. This process is particularly helpful in cases where a large number of genes of interest has been discovered. The smaller, more tractable set of "beacon" genes are used to draw attention to processes, and all the details are filled in with the entire data set as is shown below.

**Figure 5:**
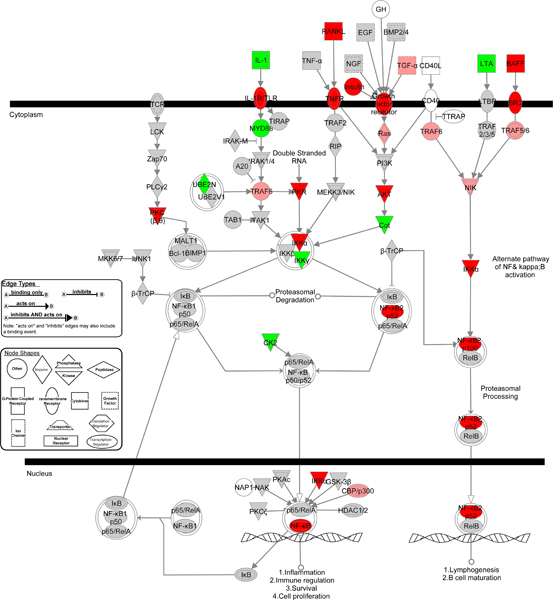
**The NFκB canonical signaling pathway from IPA**. Dark red > 3-fold increase in gene expression; light red < 3-fold increase in gene expression; dark green – > 3-fold decrease in gene expression; light green – < 3-fold decrease in gene expression; gray – unchanged gene expression; no color – gene not in array. Gene symbols with a single border represent single genes. Double border represent a complex of genes or the possibility that alternative genes might act in the pathway.

Of the 877 "off-on" genes 165 failed to map and represent unannotated genes about which little or nothing is known. The difference in annotation with the DAVID is due to IPA being curated. Of the 86 not annotated by DAVID 74 also were not annotated by IPA. Of the 714 mapped genes, 151 fit into various cell signaling processes and 133 were involved with cellular growth and proliferation. More informative were interconnected canonical pathways, many of which overlap. Any one gene may exhibit multiple functions and participate in multiple pathways. The most significant canonical pathways identified were the interconnected G-protein and NF-κB signaling networks (22 "beacon" genes combined). An NF-κB network is shown in Figure 5 with the gene expression dynamics indicated with a color code. When compared back against the set of HV genes and expressed genes, every member of the network was expressed, and many were found in the set of HV genes, which meant they showed smaller changes than the "beacons."

### Identification of potential transcriptional networks

**Figure 6:**
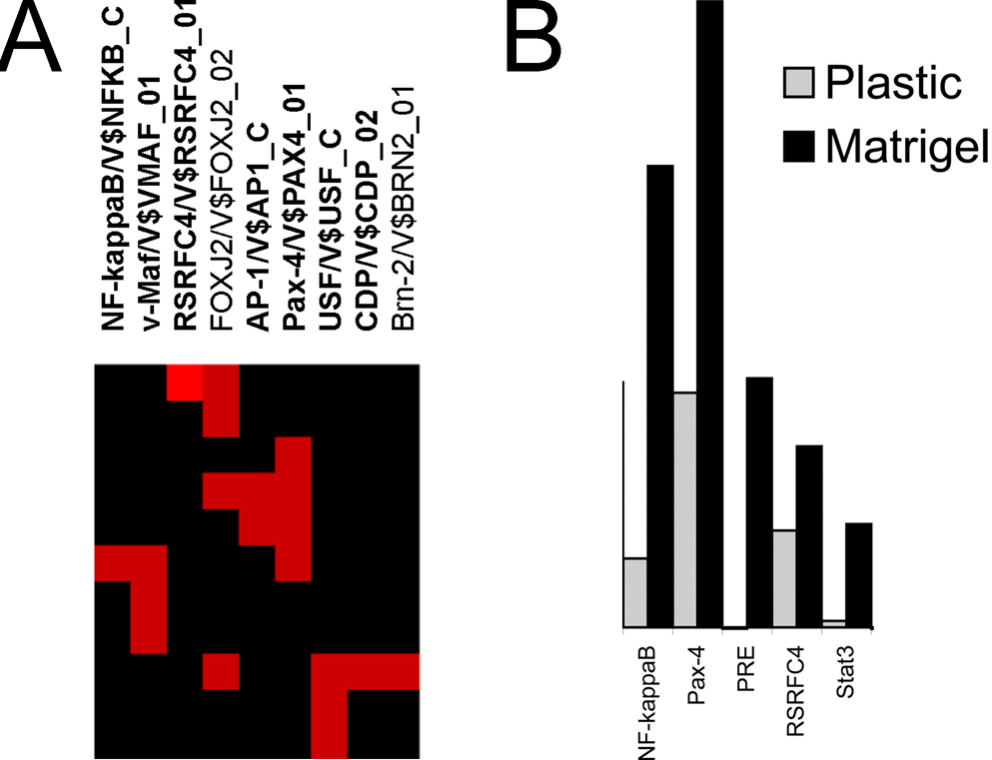
**Transcription factor activity identified *in silico *and *in vivo***. A) Example of TREs overrepresented in first ontological cluster. Several genes (vertical) share common TREs (horizontal), highlighted by red. Results were filtered to show only TREs overrepresented at p < 0.05 and FDR < 0.3. TREs in bold show a significant increase in expression on crECM compared to plastic confirmed by transcription factor array experiment. B) Example of changes in binding activity of a few TFs on plastic and crECM. Gray/black bars show binding activity on plastic/crECM, respectively.

Genes sharing similar ontologies may be regulated by one or more common transcription factors. "Off-on" genes clustered by DAVID were tested for the presence of common transcription regulatory elements (TREs) upstream of the transcribed genes in each ontological cluster by the web-based program Promoter Analysis and Interaction Network Toolset, v.3.5 (PAINT, ) [17]. PAINT queries the Transfac™ database and calculates the probability that the TREs identified in a given list of genes differ from TREs in a random sample of genes. In this case, the basis of partitioning into ontological clusters being driven by particular transcription factors was tested by comparing the TREs found in each cluster against the entire list of 21 K genes present in the microarray. This provided a map of TREs significantly overrepresented in a given cluster against a significance threshold of p < 0.05. For more reliable results filtering with a false discovery rate (FDR) < 0.3 criterion was used, when specified. An example identifying the significantly overrepresented TREs in cluster 1 is shown in Figure 6A. The over-represented TREs are also summarized in Table 1 by cluster. The probability of a random collection of genes sharing a common TRE is less than 0.05. Thus the finding that a set of genes contains common TREs or fit into known networks supports that they are neither randomly selected by chance, nor the product of technical error to within the limits of statistical testing.

The predictions of PAINT were tested using an independent experimental assay that measured the DNA binding activity of transcription factors using TranSignal Protein/DNA Combo Arrays. This array allows estimation of binding activity of 345 TFs. We found a number of up-regulated transcription factors on crECM. The bar diagram in Figure 6B compares the activity of selected transcription factors in cells grown on plastic vs. crECM. The majority of transcription factors that showed large changes in activity were also identified by PAINT as driving up- or down-regulated clusters of genes, the results are shown in Table 1 with TREs confirmed by Panomics array highlighted in bold. Depending on stringency, between 5 and 13 transcription factors were shut off and between 25 and 40 were activated by the crECM. Supplemental table S1 in Additional file 1 shows DNA binding activity of all transcription factors in cells grown on plastic or crECM. Most of the changes were "off-on," as was observed for the mRNAs of the downstream genes. Some transcription factors were active in cells growing under both conditions.

## Conclusion

In this study we present a self-guided approach for analyzing a complex biological change by microarrays and illustrate its use to describe the complex change in gene expression that occur when papilloma cells are placed on a crECM. The flowchart of each step of this approach is shown in Figure 1. We also confirm the validity of the integrated approach by independent verification of the predictions of transcriptional regulatory networks. With a very complex biological system mobility more than 2000 genes identified as significantly varying, we show that the essential elements of the change in the large scale picture of the biology can be captured in a smaller subset of "beacon" genes. Analysis of this more concise set of "beacons" facilitates mapping the gene expression dynamics onto known processes [18]. We wish to emphasize that the resulting biological picture does not derive solely from indentifying only a few key genes. This approach also requires that all members of a pathway be expressed, which is determined by comparing putative networks or canonical pathways against the entire dataset of expressed genes. Genes showing smaller changes than shown by the "beacons" are identified against the set of HV genes.

The approach also is statistically robust. Expression is judged against the uncertainty of the zero point, and the threshold can be selected either to minimize false negatives or false positives. The need for replicates, and therefore the cost of experiments, is minimized using a global F-test against the variance of the system as a whole with a p-value standard of 1/N that minimizes false positive identification of significant genes. The HV gene approach is best suited to providing an overall description for hypothesis generation with multiple biological variables as opposed to hypothesis-testing in a two-state system (e.g. treated and control).

In summary, this article demonstrates an approach to microarray analysis that organizes the findings into a biologically based model that should in turn, facilitate generation and testing of hypotheses because the analysis itself is structured around the properties of biological system. In this case, the findings suggest that G-protein signaling plays a major role in the modulation of phenotype by crECM, that the cells are differentiated and acquire specialized functions (e.g. immune function and transmembrane proteins) and that several transcription factors regulate the process.

## Methods

The RT4 bladder transitional cell papilloma cell line (American Type Culture Collection, Manassas Virginia) was cultured on plastic and on cancer-remodeled ECM, Matrigel™ (crECM), and RNA was isolated as described previously using the RNAeasy kit (QIAGEN Inc., Valencia, CA) [8,19].

Microarray data were obtained using a spotted array from cells cultured on plastic (two arrays) and across 9 days time course of growth on crECM, as previously described [9]. Cy3 labeled cDNA was synthesized and hybridized onto glass arrays spotted with 22,464 long oligos (~70 mers) from the UniGene database of functionally known genes.

Expression data were normalized to the variability around the zero point, as described previously [5,7]. Genes were considered to be expressed if their expression normalized to the S.D. of zero point exceeded 3.0 (p < 0.001). The arrays were then globally adjusted to each other by robust linear regression. Genes expressing higher variability than the technical variability of the system (hypervariable, or HV genes) were identified as described previously [4] and as shown in Figure 3B. Two thresholds were used. HV genes showed relative standard deviations > 3.8 SD above that of the population mode (p < 6 .7 × 10^-5^). This value is 1/N, where N is the number of expressed genes (~15,000). A more stringent criterion of > 5 SD (p < 2.87 × 10^-7^) was also used to identify a subset of very HV, or "beacon" genes. Their expression profiles were clustered by the Cluster 3.0 program [20]. Hierarchical clustering identified major patterns of gene expression changes; further clustering of selected "beacon" genes was done using K-means clustering, k = 3, which was selected as described below. Significantly over-represented gene ontologies were identified using the Database for Annotation, Visualization and Integrated Discovery (DAVID, ) [16]. Biologically relevant networks were assembled from identified clusters and groups of common genes by using Ingenuity Pathways Analysis (IPA, ). Each gene identifier was mapped to its corresponding gene object in the Ingenuity Pathways Knowledge Base. Genes were not weighted by expression levels, and biological networks were built on this assumption. Analysis of common TREs shared by genes in each ontological cluster was performed by using the web-based program PAINT [17] against whole list of genes in microarray.

Additional assessment of the DNA binding activity of transcription factors using TranSignal Protein/DNA Combo Arrays (spin column version, # MA1215, Panomics, Redwood City, CA) was conducted according to the manufacturer's protocol. Briefly, cell nuclear extracts were incubated with biotin-labeled oligonucleotides that possess consensus DNA binding sites for 345 transcription factors. The protein bound probes were isolated by using a spin column and then hybridized to the DNA/protein array. After the DNA/protein array was washed, the array was incubated with detection solution and images of the chemiluminescent signal were captured using an Alpha Imager (Alpha Innotech, San Leandro, CA) and quantitated by using AlphaEase software and standardized against biotinylated DNA spots on the membrane. The results are linear between 0 and 100 units. In order to detect low expression transcription factor activity, two exposure times were used, 8 and 25 min. Values were adjusted for exposure so that all values were measured within the linear range of the assay. A histogram of expression data was plotted and was found to be bimodal, with one mode centered about zero. Background subtraction was performed by calculating the standard deviation of this distribution and subtracting 3 standard deviations above the mode from all expression values, approximately 6 units.

## List of abbreviations used

cDNA – complementary deoxyribonucleic acid; crECM – cancer-remodeled extracellular matrix; DAVID – Database for Annotation, Visualization and Integrated Discovery; DNA – deoxyribonucleic acid; ECM – extracellular matrix; FDR – false discovery rate; HV – hypervariable genes; IPA – Ingenuity^© ^Pathway Analysis; mRNA -messenger ribonucleic acid; PAINT – Promoter Analysis and Interaction Network Toolset; SD – standard deviation; TF – transcription factor; TRE – transcription regulatory element; SSC – Saline-Sodium Citrate

## Competing interests

The authors declare that they have no competing interests.

## Supplementary Material

Additional file 1 Supplemental Table S1. Activity of 345 transcription factors in cells grown on Matrigel and on Plastic. Values were obtained as described in Methods and are arbitrary units, but are normalized to protein content and exposure time against an assay standard of biotinylated DNA. Click here for file
