# Supplementary material for: From microarray to biology: an integrated experimental, statistical and in silico analysis of how the extracellular matrix modulates the phenotype of cancer cells
Source: BMC Bioinformatics. 2008 Aug 12;9(Suppl 9):S4. doi: 10.1186/1471-2105-9-S9-S4 (PMC2537575; doi:10.1186/1471-2105-9-S9-S4)
Supplement: Additional file 1 — Supplemental Table S1. Activity of 345 transcription factors in cells grown on Matrigel and on Plastic. Values were obtained as described in Methods and are arbitrary units, but are normalized to protein content and exposure time against an assay standard of biotinylated DNA. [file 1471-2105-9-S9-S4-S1.doc]

Supplemental Table S1. Activity of 345 transcription factors in cells grown on Matrigel and on Plastic. Values were obtained as described in Methods and are arbitrary units, but are normalized to protein content and exposure time against an assay standard of biotinylated DNA.

| **Transcription factor** | **Plastic** | **Matrigel** | **TF description** | **TF change** |
| --- | --- | --- | --- | --- |
| AP-2 | -0.51 | 142.46 | activating enhancer binding protein 2 | off-ON |
| CREB | -0.51 | 89.19 | CREB1: cAMP responsive element binding protein 1 | off-ON |
| CDP | -0.48 | 71.99 | CCAAT displacement protein | off-ON |
| Brn-3 | -0.52 | 68.38 | POU4F1: POU domain, class 4, transcription factor 1 | off-ON |
| PRE | -0.37 | 66.81 | Progesterone receptor | off-ON |
| NFATc | -0.28 | 65.76 | NFATC: nuclear factor of activated T-cells, cytoplasmic, | off-ON |
| AP1 | -0.57 | 61.66 | Activator protein 1 including Fos, FosB, Fra1, Fra2, Jun, JunB | off-ON |
| LR1 | -0.14 | 45.46 | LR1 is a 106-kDa sequence-specific DNA-binding protein first identified as a potential regulator of immunoglobulin class switch recombination in B lymphocytes | off-ON |
| CEA | -0.07 | 32.24 | carcinoembryonic antigen gene | off-ON |
| GATA | -0.54 | 24.11 | GATA: GATA binding protein (globin transcription factor ) | off-ON |
| GAS/ISRE | -0.52 | 23.73 | interferon activated factors | off-ON |
| FAST-1 | -0.51 | 23.43 | FOXH1: forkhead box H1 | off-ON |
| CPE | -0.30 | 20.02 | Cap proximal element | off-ON |
| AML1 | -0.01 | 19.96 | RUNX1: runt-related transcription factor 1 (acute myeloid leukemia 1; aml1 oncogene) | off-ON |
| ARE | -0.56 | 16.50 |  | off-ON |
| GATA-3 | -0.04 | 12.62 | GATA binding protein3 (globin transcription factor 3) | off-ON |
| AAF | -0.34 | 11.27 | an IFN-gamma-regulated DNA-binding factor | off-ON |
| NF-1 | -0.22 | 11.05 | NF-1nuclear factor I | off-ON |
| SRY | -0.40 | 9.86 | testis determining factor | off-ON |
| Tax/CREB | -0.40 | 9.23 | TAX &CREB complex-responsive protein | off-ON |
| Ikaros | -0.30 | 8.12 |  | off-ON |
| AP2 | -0.28 | 7.20 | activator protein 2 | off-ON |
| PPARa | -0.30 | 6.77 | peroxisome proliferator activated receptor | off-ON |
| FKHR | -0.17 | 4.41 | forkhead box O1A (rhabdomyosarcoma) human | off-ON |
| MTF(1) | -0.46 | 4.01 | MRE-binding transcription factor-1 | off-ON |
| IRF-1 | -0.45 | 3.79 | IRF1: interferon regulatory factor 1 | off-ON |
| MEF-2 | -0.51 | 3.60 | MEF-2Myocyte enhancing factor2 | off-ON |
| MTB-Zf | -0.30 | 3.57 | cis- regulatory element (MTE) binding protein | off-ON |
| E2 | -0.23 | 2.54 | BPV-1, bovine papilloma virus type 1 | off-ON |
| NRF-1 | -0.36 | 1.84 | nuclear respiratory factor 1; | off-ON |
| EIL1/2/3 | -0.59 | 1.81 | ETHYLENE-INSENSITIVE3-LIKE1/2/3 | off-ON |
| v-Maf | -0.04 | 1.78 | v-maf musculoaponeurotic fibrosarcoma oncogene homolog (avian) | off-ON |
| TTF1 | -0.20 | 1.46 | Thyroid-specific enhancer. Homog of Drosophila NK2 factor, and mammalian Nkx family; site in promoters of eight thyroid-specific genes; also regulates pulmonary-specific gene expression | off-ON |
| NPAS2 | -0.42 | 1.32 | neuronal PAS domain protein 2 | off-ON |
| GAS | -0.28 | 1.00 | gamma-interferon activation site | off-ON |
| HiNF-B/H1TF1 | -0.23 | 0.65 | Human H1 Histone Gene Promoter CCAAT Box Binding Protein   HiNF-B Is a Mosaic Factor | off-ON |
| TEF1(AP5) | -0.20 | 0.56 | activator; function depends on a highly limiting cofactor.  interference with TEF-1 / TFIID interaction by a B-cell specific factor | off-ON |
| Oct--1 | -0.33 | 0.21 | POU2F1: POU domain, class 2, transcription factor 1 | off-ON |
| TCE | -0.48 | 0.05 | Transforming Growth Factor ß1control element | off-ON |
| EGF BP | -0.40 | 0.02 |  | off-ON |
| SPERM1 | 13.99 | -0.44 | A Pou domain gene transiently expressed immediately   prior to meiosis I in the male germ cell | ON-off |
| HFH-11B/11a | 8.73 | -0.82 |  | ON-off |
| PEPCK promoter | 6.35 | -1.55 |  | ON-off |
| LF-A1(2) | 3.97 | -2.20 | Liver-specific transcription factor | ON-off |
| Freac-7 | 1.09 | -1.58 | forkhead box L1 | ON-off |
| C/EBPa/g | 0.74 | -2.31 | CCAAT/enhancer binding protein alpha,gamma | ON-off |
| MyoD | 0.36 | -1.47 | myogenic factor D | ON-off |
| MyoG | 0.13 | -0.95 | myogenic factor G | ON-off |
| Pit 1 | 0.12 | -0.71 | POU1F1: POU domain, class 1, (Pit1, growth hormone factor 1) | ON-off |
| EGR2 BP | 0.09 | -1.14 | Early growth factor2 binding protein | ON-off |
| Pax2/5/8 | 0.06 | -2.04 |  | ON-off |
| MEF-3 | 0.01 | -2.04 | myelin gene expression factor 3 | ON-off |
| PPARr | 0.01 | -2.82 | peroxisome proliferator activated receptor | ON-off |
| MZF1(1) | 0.04 | 6.44 | myeloid zinc finger 1 | level |
| PPAR | 0.06 | 14.87 | PPAR: peroxisome proliferative activated receptor | level |
| KKLF | 0.09 | 12.13 | Kruppel-like factor | level |
| ALF1B | 0.15 | 3.90 | murine leukemia virus | level |
| C/EBPg | 0.19 | 1.16 | CCAAT/enhancer binding protein gamma | level |
| ADD1 | 0.22 | 12.97 | sterol regulatory element binding transcription factor | level |
| ANG-IRE | 0.22 | 2.00 | Angiotensinogen(ANG) insulin-response factor | level |
| TxREF NF III | 0.24 | 2.00 |  | level |
| NZF-3 | 0.27 | 32.62 | neural zinc finger factor 3 | level |
| c-Myb | 0.33 | 95.72 | MYB: v-myb myeloblastosis viral oncogene homolog (avian) | level |
| LyF-1(2) | 0.38 | 14.63 | lymphoid transcription factor, Ikaros | level |
| TIF1 | 0.39 | 3.30 | traT/EBP, thyroid-specific enhancer-binding protein, thyroid nuclear nscriptional intermediary factor 1 | level |
| MZF1 | 0.54 | 6.34 | zinc finger protein 42 (myeloid-specific retinoic acid- responsive) | level |
| AF1 | 0.61 | 1.00 | NR2F2: nuclear receptor subfamily 2, group F, member 2 | level |
| ETF | 0.61 | 80.66 | DNA Binding Factor That Represses Transcription | level |
| SIF2 | 0.61 | 5.93 | SI promoter 2 | level |
| Ets | 0.64 | 82.50 | ETS: v-ets erythroblastosis virus E26 oncogene homolog (avian) | level |
| Stat1 p84/p91 | 0.68 | 10.83 | STAT1: signal transducer and activator of transcription 1, 84kDa/91kDa | level |
| HFH-8 | 0.77 | 16.58 |  | level |
| ICSBP | 0.77 | 5.77 | interferon consensus sequence binding protein | level |
| C/EBP | 0.85 | 96.35 | CEBPA: CCAAT/enhancer binding protein (C/EBP), alpha | level |
| TREF1_2 | 1.19 | 4.63 |  | level |
| ATF-adelta | 1.25 | 7.72 |  | level |
| NFIL-2 | 1.72 | 19.26 | 5' upstream activating sequences (UAS) of the human IL-2 gene | level |
| KPF1 | 1.75 | 8.37 | Keratinocyte-Specific Transcription Factor | level |
| Stat3 | 1.75 | 27.74 | STAT3: signal transducer and activator of transcription 3 | level |
| Ets-1/PEA3 | 1.87 | 43.59 | ETS-domain transcription factor pea3 | level |
| NFkB(2) | 2.26 | 84.48 | NFkB | level |
| GATA-6 | 2.50 | 2.65 | GATA binding protein 6 (globin transcription factor 6) | level |
| AFP1 | 2.88 | 36.66 | alpha-fetoprotein | level |
| Skn | 2.91 | 6.93 | octamer-binding site in epidermis (POU domain factor) | level |
| RFX1 2 3 (1) | 2.93 | 3.06 |  | level |
| HNF-4a2/1 | 3.02 | 8.48 | hepatocyte nuclear factor-4 | level |
| CCAAT | 3.03 | 19.26 | CCAAT binding protein | level |
| p300 | 3.81 | 79.11 | p300 coactivator | level |
| HOX4C | 3.83 | 8.86 | homeo box4C | level |
| AP4 | 4.27 | 32.73 | activator protein 4 binding protein | level |
| ERE | 4.32 | 118.56 |  | level |
| kBF-A | 4.85 | 29.42 | kappa immunoglobulin enhancer binding protein | level |
| GATA-4 | 5.07 | 12.00 | GATA binding protein 4 (globin transcription factor 4) | level |
| SIE | 5.17 | 73.42 | serum inducible element responsive factor | level |
| CBF | 5.39 | 75.21 | mouse CCAAT-binding factor, CP1 (human, rat); NF-Y | level |
| RXR(DR-1) | 5.51 | 38.77 | RXR: retinoid X receptor | level |
| Fra-1/JUN | 5.63 | 7.37 | Fos-related antigen | level |
| Tf-LF | 5.83 | 32.13 | a liver-specific factor | level |
| RIPE3a1 | 6.06 | 12.81 | rat insulin promoter element 3(RIPE3)can confer either positive regulation in insulin-producing cells | level |
| c-myb BP | 6.33 | 91.69 | c-myb enhancer binding protein | level |
| LyF(1) | 6.38 | 72.37 | lymphoid transcription factor, Ikaros | level |
| Myc-Max | 6.87 | 48.88 | myc-associated factor X | level |
| ARP | 6.96 | 7.80 | COUP-beta; apolipoprotein AI regulatory protein; NR2F2 | level |
| PRDII-BF1 | 7.11 | 81.88 | alphaA-crystallin binding protein I | level |
| EKLF(2) | 7.23 | 33.38 | erythroid Kruppel-like factor gene | level |
| T3R | 7.27 | 47.01 | c-ErbA; thyroid hormone receptor | level |
| XBP-1 | 7.72 | 24.06 | X-box binding protein 1 | level |
| Smad3/4 | 8.32 | 64.24 | MADH3/4: MAD, mothers against decapentaplegic homolog3/4 | level |
| MAZ | 8.43 | 43.62 | MYC-associated zinc finger protein (purine-binding transcription factor) | level |
| RB | 8.64 | 16.01 | retinoblastoma tumor suppressor protein | level |
| Gfi-1 | 9.68 | 50.83 | growth factor independent 1 | level |
| TFE3-L | 10.41 | 15.76 | TFE3-L is an approximately 3-fold stronger activator than TFE3-S;   cooperating with ITF-1, TFE3 can mediate lymphoid-specific   activation through combined elements (such as muE5   and muE3 in the IgH enhancer) | level |
| ACPBP | 10.81 | 24.68 |  | level |
| Pax3 | 11.48 | 27.36 | paired box gene 3 | level |
| NF-1/L | 11.82 | 20.70 | NF1/L; RPF-A (hamster); NF-1A1.1 (chick); NF-I/L | level |
| HFH | 12.14 | 12.92 | HNF-3/Fkh Homolog 1/2; HFH | level |
| HNF-3beta | 12.15 | 60.12 | hepatic nuclear factor 3 | level |
| C/EBPa(1) | 12.46 | 50.31 |  | level |
| HNF-1A | 12.86 | 42.53 | hepatocyte nuclear factor 1 | level |
| GRE | 13.18 | 72.61 |  | level |
| NFE-6/CP1 | 13.71 | 74.32 | A highly conserved enhancer downstream of the human   MLC1/3 locus is a target for multiple myogenic determination factors | level |
| E2F-2 | 14.60 | 177.09 | E2F1: E2F transcription factor 1 | level |
| NF-Y | 14.60 | 98.16 | NF-Y, also known as CBF (CCAAT-binding factor), was first identified as a protein that binds to the Y box of MHC (major histocompatibility complex) class II E promoters | level |
| p53(2) | 15.74 | 54.92 | Tumor protein p53 | level |
| CP1 | 15.83 | 18.01 | CBF; NF-Y | level |
| lactoferrin BP | 16.22 | 29.37 |  | level |
| PCF | 17.62 | 17.74 | Promoter-linked coupling element | level |
| USF-1 | 17.64 | 77.70 | USF: upstream transcription factor | level |
| NFkB | 18.49 | 123.79 | nuclear factor of kappa light polypeptide gene enhancer in B-cells 1 | level |
| MRE | 19.20 | 103.12 | Metal response factor | level |
| H4TF-1 | 19.71 | 42.53 | Histone 4 binding protein | level |
| MDBP(1) | 20.49 | 35.90 | methylated DNA-binding protein | level |
| E4BP4 | 21.55 | 89.08 | nuclear factor, interleukin 3 regulated | level |
| NF-1(2) | 22.77 | 76.65 | CTF; NF-I; TGGCA-binding protein | level |
| RREB | 23.76 | 61.91 | Ras-responsive element binding protein 1 | level |
| XRE | 24.02 | 97.00 |  | level |
| NF-Atp | 25.73 | 26.39 | NFII-a; NF-ATc2; NF-IL2E; NF-AT1; | level |
| RSRFC4 | 25.96 | 48.55 | MADS box transcription enhancer factor 2 | level |
| SRF(2) | 26.36 | 26.95 | serum response factor; CArG-binding factor; CBF (3) (mouse); p67; p67SRF | level |
| ISRE | 26.70 | 93.39 | interferon-a stimulated response element | level |
| PUR | 27.32 | 67.49 | Pur factor | level |
| Pax-5 | 28.62 | 93.99 | PAX5: paired box gene 5 (B-cell lineage specific activator protein) | level |
| AIC, CBF | 29.52 | 32.24 |  | level |
| Elk1 | 30.04 | 39.80 | member of ETS oncogene family | level |
| p53 | 31.66 | 68.79 | p53 tumor protein | level |
| LF-A1 | 32.16 | 86.10 | liver-specific TF | level |
| TR(DR-4) | 32.26 | 80.14 | thyroid hormone receptor | level |
| PEBP2 | 32.91 | 46.41 | polyoma enhancer binding protein | level |
| Pax5(2) | 36.10 | 63.29 | Pax5 is required for the early stages of B-cell differentiation, Pax-5DNA-binding transcription factor | level |
| NF-Y | 36.21 | 70.33 | NF-Y, also known as CBF (CCAAT-binding factor), was first identified as a protein that binds to the Y box of MHC (major histocompatibility complex) class II E promoters | level |
| EVI-1 | 36.62 | 73.04 | ecotropic viral integration site 1(zinc finger oncogene) | level |
| HiNF | 37.94 | 133.14 | histone gene transcription   factors | level |
| SP1 ASP | 38.12 | 118.92 | Sp1 transcription factor | level |
| Stat4 | 40.34 | 86.86 | STAT4: signal transducer and activator of transcription 4 | level |
| LyF-1(3) | 40.87 | 112.96 | lymphoid transcription factor, Ikaros | level |
| VDR(DR-3) | 41.08 | 83.77 | VDR: vitamin D (1,25- dihydroxyvitamin D3) receptor | level |
| NF-E2(2) | 41.74 | 62.53 | NF-E2 protein | level |
| HIF-1 | 41.91 | 105.21 | hypoxia-inducible factor 1 | level |
| EGR | 42.15 | 199.71 | early growth response | level |
| Snail | 42.15 | 56.38 | zinc-finger transcription factor Snail | level |
| Afxh (Foxo4) | 42.43 | 121.08 | Member of the forkhead family | level |
| RAR(DR-5) | 42.46 | 101.52 | RAR: retinoic acid receptor | level |
| MEF-1 | 42.83 | 133.49 | MEF-1Myocyte enhancing factor1 | level |
| GATA-1/2 | 43.01 | 137.42 | GATA binding protein 1/2 (globin transcription factor) | level |
| TR | 43.05 | 71.09 | thyroid hormone receptor | level |
| RFX1/2/3 | 43.36 | 69.55 | a transactivator of hepatitis B virus enhancer   I, belongs to a novel family of homodimeric and heterodimeric   DNA-binding proteins | level |
| NF-E2 | 43.73 | 135.09 | NFE2: nuclear factor (erythroid-derived 2), 45kDa | level |
| Stat1/Stat3 | 44.49 | 89.19 | signal transducer and activator of transcription 1/3 | level |
| Pax6 | 46.86 | 123.28 | Pax-6 plays an essential role in the development and   function of glucagon-producing cells in both pancreatic   and intestinal endodermal lineages | level |
| CEF2 | 47.70 | 110.30 | cTnC (Slow/Cardiac Troponin C ) | level |
| MT-Box | 47.74 | 129.92 | identified in differentiation system, located in hTERT promotoer | level |
| GBF1/2/3/HY5 | 48.39 | 63.67 |  | level |
| SAA | 49.76 | 94.15 |  | level |
| GATA-1 | 49.94 | 123.93 | GATA binding protein 1 (globin transcription factor 1) | level |
| GAG | 50.74 | 139.62 | amyioid precursor proten (APP) regulatory element | level |
| c-Rel | 51.69 | 120.89 | NFkB p75kDa protein | level |
| E12 | 52.07 | 100.74 | E2A immunoglobulin enhancer binding factors E12 | level |
| RORE | 53.14 | 75.59 | RAR-related orphan receptor | level |
| ODC | 53.46 | 69.55 | ODC (ornithine decarboxylase | level |
| p53 | 53.52 | 141.19 | p53 tumor protein | level |
| GATA-2 | 53.92 | 179.15 | GATA binding protein 2 (globin transcription factor 2) | level |
| Myb(2) | 54.24 | 81.52 | v-myb myeloblastosis viral oncogene homolog (avian) | level |
| PBGD BP | 54.63 | 85.32 |  | level |
| NF-E1 (YY1) | 54.80 | 138.72 | YY1 transcription factor | level |
| MBP-1 | 55.11 | 94.80 |  | level |
| E4F, ATF | 55.24 | 104.15 | E4F transcription factor 1 | level |
| HFH-1 | 56.12 | 128.59 | winged helix/forkhead transcription factor | level |
| AP3 | 56.30 | 138.34 | activator protein 3 | level |
| HOXD8 9 10 | 56.67 | 155.93 | homeo box D8 | level |
| Pax8 | 57.72 | 138.43 | paired box gene 8 | level |
| XBP1(2) | 59.15 | 103.15 | X-box binding protein 1; TREB-5; hXBP-1 | level |
| Beta-RE | 59.45 | 127.97 | retinoic acid receptor beta | level |
| PU.1 | 59.51 | 100.14 | Spi-1; NF-JB; B1. acts as an activator or as a repressor;   after phosphorylation at Ser-148 by CK II, PU.1 recruits   NF-EM5 to bind to DNA resulting in pronounced transcriptional   activation; PU.1 interferes with the commitment of erythroblast to differentiate;   activated by proviral integration of SFFV (anemia-   or polycythemia-inducing strains) in 95% of leukemic cell clones | level |
| EGR1 | 60.82 | 115.07 | Early growth factor1 | level |
| Antioxidant RE | 61.22 | 156.12 | Antioxidant responsive factor | level |
| L-III BP | 61.35 | 157.83 | pyruvate kinase L gene binding protein III (hepatocyte specific) | level |
| TFEB | 61.43 | 104.31 | TFEB is closely related to TFE3   leucine zipper: essential for dimerization [4];   free in solution: tetramerization [4]; | level |
| MZF1(2) | 61.69 | 125.31 | myeloid zinc finger 1 | level |
| NCAM BP | 62.21 | 97.57 |  | level |
| Sp1 | 62.29 | 152.87 | SP1: Sp1 transcription factor | level |
| PAX1 | 62.42 | 113.77 |  | level |
| ADR1 | 62.85 | 143.17 | alcohol dehydrogenase regulatory protein | level |
| Pax4 | 62.96 | 168.01 | paired box gene 4 | level |
| ISRE | 63.49 | 141.65 | interferon-a stimulated response element | level |
| CYP1A1 | 63.80 | 113.82 | cytochrome P450-c promoter region | level |
| pax2 | 64.00 | 94.31 | Pax-2DNA-binding transcription factor | level |
| YB1 | 65.25 | 140.29 | DbpB; EFIA | level |
| NF-4FA | 67.46 | 123.85 | 4F2 Heavy-Chain Gene enhancer element | level |
| NF-A3 | 67.48 | 69.33 | Nuclear factor A3 | level |
| Surf-2(2) | 67.51 | 155.93 | Surfeit promoter region | level |
| HFH-2 | 67.59 | 114.72 | forkhead box D3 | level |
| CCAC | 68.15 | 161.02 | CCAC binding protein | level |
| transferrin BP | 69.05 | 100.19 |  | level |
| E47 | 69.18 | 169.61 | E2A immunoglobulin enhancer binding factors E12/E47 | level |
| EBP40_45 | 70.63 | 144.36 |  | level |
| HFH-3 | 73.76 | 175.95 | forkhead box I1 | level |
| WT1(1) | 75.67 | 115.39 | Wilms tumor 1 | level |
| WT1(2) | 78.22 | 134.20 | Wilms tumor 1 | level |
| HOXD9 10 | 79.01 | 139.97 | HOXD9,10 | level |
| TEF1 | 80.30 | 172.84 | activator; function depends on a highly limiting cofactor.  interference with TEF-1 / TFIID interaction by a B-cell specific factor | level |
| Thy-1BP | 80.80 | 160.24 | Thy-1binding protein | level |
| CTCF | 82.91 | 122.74 | CCCTC binding factor | level |
| PARP | 83.47 | 165.09 |  | level |
| PYR | 85.08 | 148.21 | pyrimidine-rich domain binding factor | level |
| AhR/Arnt | 86.39 | 189.64 | aryl hydrocarbon receptor/aryl hydrocarbon receptor nuclear translocator binding element | level |
| HMG | 87.66 | 161.46 | high mobility group proteins | level |
| MSP1 | 91.45 | 158.18 | the sequences are the same as SAA(without SP1 binding site ) | level |
| MUSF1 | 97.34 | 151.97 |  | level |
| WAP BP | 75.62 | 49.01 |  | level |
| Freac-2 | 54.19 | 10.97 | forkhead box F2 (mouse) | level |
| RVF | 40.03 | 14.41 | EcoRV factor on the neu promoter | level |
| EKLF(1) | 32.85 | 22.27 | erythroid Kruppel-like factor gene | level |
| Pax-6 | 30.70 | 1.51 |  | level |
| Freac-4 | 29.98 | 28.15 | forkhead box D1 | level |
| CREB2 | 29.58 | 8.31 | cyclic AMP response element binding protein 2. An ATF/CREB transcription   factor that can negatively regulate transcription   from the cAMP response element | level |
| PTF1 | 26.31 | 14.36 | pancreas specific transcription factor | level |
| LXRE1 | 19.76 | 14.55 | nuclear receptor subfamily 1, group H, member 2 | level |
| TFIID | 14.99 | 2.65 | TBP: TATA box binding protein | level |
| CACC | 14.43 | 6.50 | CACC binding protein | level |
| LF-A2 | 10.49 | 9.23 | liver-specific factors stimulate in   vitro transcription from the human alpha1-antitrypsin promoter | level |
| N-ras BP | 8.92 | 7.72 | N-ras promoter region | level |
| COUP-TF | 8.17 | 3.22 | COUP-TF; EAR3; NR2F1; COUP-alpha | level |
| ATF/CRE | 7.36 | 0.59 | Activating transcription factor | level |
| WT1(3) | 7.31 | 4.14 | Wilms tumor 1 | level |
| LH2/Lim1 | 4.56 | 1.49 | LIM homeobox 2 | level |
| HSE | 2.47 | 1.19 | heat shock transcription factor | level |
| DE I | -0.01 | -2.04 |  | NotExpr |
| TFE3 | -0.02 | -2.63 |  | NotExpr |
| Oct-4/Oct3 | -0.02 | -4.64 |  | NotExpr |
| CEF1 | -0.02 | -4.50 |  | NotExpr |
| SRE | -0.08 | -0.71 |  | NotExpr |
| NFAT-1 | -0.13 | -5.99 |  | NotExpr |
| SSAP | -0.14 | -4.85 |  | NotExpr |
| MDBP(2) | -0.14 | -2.90 |  | NotExpr |
| Freac-2 | -0.14 | -5.42 |  | NotExpr |
| Fkhr | -0.14 | -3.01 |  | NotExpr |
| IRF-1, IRF-2 | -0.17 | -0.41 |  | NotExpr |
| HOXD8(2) | -0.17 | -3.04 |  | NotExpr |
| Stat5b | -0.19 | -2.52 |  | NotExpr |
| CTEF-CRE | -0.19 | -0.68 |  | NotExpr |
| CREB-BP1 | -0.22 | -4.85 |  | NotExpr |
| AREB6 | -0.22 | -0.95 |  | NotExpr |
| myc-PRF | -0.25 | -6.59 |  | NotExpr |
| Pur-1 | -0.27 | -5.40 |  | NotExpr |
| myc-CF1 | -0.27 | -5.86 |  | NotExpr |
| LSF | -0.27 | -4.77 |  | NotExpr |
| v-rel 50-55K | -0.28 | -1.39 |  | NotExpr |
| MyTI | -0.28 | -3.69 |  | NotExpr |
| LF-B2 | -0.28 | -6.56 |  | NotExpr |
| M-globin factor B1 | -0.30 | -4.77 |  | NotExpr |
| HNF-3(a, b, r) | -0.30 | -6.40 |  | NotExpr |
| Nkx-2.5 | -0.31 | -3.09 |  | NotExpr |
| HNF-4 & COUPTF | -0.31 | -5.45 |  | NotExpr |
| HNF-4 | -0.31 | -4.39 |  | NotExpr |
| CSBP | -0.31 | -1.09 |  | NotExpr |
| Stat5 | -0.33 | -2.09 |  | NotExpr |
| CRE | -0.33 | -5.40 |  | NotExpr |
| ATF-a | -0.33 | -5.99 |  | NotExpr |
| ATF2 | -0.33 | -2.44 |  | NotExpr |
| Smad SBE | -0.34 | -3.39 |  | NotExpr |
| ORE | -0.34 | -0.55 |  | NotExpr |
| CdxA/NKX2 | -0.34 | -6.64 |  | NotExpr |
| CD28RC(2) | -0.34 | -3.31 |  | NotExpr |
| BZP | -0.34 | -6.24 |  | NotExpr |
| URE | -0.36 | -3.93 |  | NotExpr |
| TCF/LEF | -0.36 | -1.52 |  | NotExpr |
| PRDI-BFc | -0.36 | -4.88 |  | NotExpr |
| Isl-1 | -0.36 | -4.96 |  | NotExpr |
| HLF | -0.36 | -4.61 |  | NotExpr |
| CP1 CTF CBTF | -0.36 | -3.07 |  | NotExpr |
| NF-Gma | -0.37 | -1.25 |  | NotExpr |
| NF-Atx | -0.37 | -7.02 |  | NotExpr |
| GKLF | -0.37 | -3.53 |  | NotExpr |
| Cdx2 | -0.37 | -1.87 |  | NotExpr |
| PPUR(2) | -0.39 | -2.12 |  | NotExpr |
| MBP-1 | -0.39 | -5.80 |  | NotExpr |
| LCR-F1 | -0.40 | -0.68 |  | NotExpr |
| KTP1 | -0.40 | -6.18 |  | NotExpr |
| HEN1 | -0.40 | -3.58 |  | NotExpr |
| ACF | -0.40 | -4.10 |  | NotExpr |
| PO-B | -0.42 | -5.23 |  | NotExpr |
| GATA1(2) | -0.43 | -7.08 |  | NotExpr |
| CD28RC(1) | -0.43 | -1.85 |  | NotExpr |
| ZNF174 | -0.45 | -5.07 |  | NotExpr |
| Tat | -0.45 | -3.91 |  | NotExpr |
| HOXD8(1) | -0.45 | -5.23 |  | NotExpr |
| Elf | -0.45 | -4.85 |  | NotExpr |
| COUP-TF(2) | -0.45 | -6.35 |  | NotExpr |
| X2BP | -0.46 | -4.75 |  | NotExpr |
| SIF3 | -0.46 | -3.58 |  | NotExpr |
| SIF1 | -0.46 | -5.97 |  | NotExpr |
| PEBP | -0.46 | -4.45 |  | NotExpr |
| CP1B | -0.46 | -6.70 |  | NotExpr |
| p55 | -0.48 | -3.58 |  | NotExpr |
| HOXA4 | -0.48 | -6.89 |  | NotExpr |
| HFH-8/3 LUN | -0.48 | -4.50 |  | NotExpr |
| c-Ets-1 | -0.48 | -7.08 |  | NotExpr |
| AP3 | -0.48 | -4.72 |  | NotExpr |
| Stat6 | -0.49 | -8.05 |  | NotExpr |
| PTF1-beta | -0.49 | -3.34 |  | NotExpr |
| MEF-2a | -0.49 | -6.21 |  | NotExpr |
| HiNF-A | -0.49 | -3.58 |  | NotExpr |
| EBP-80 | -0.49 | -4.77 |  | NotExpr |
| E12/E47 | -0.49 | -1.31 |  | NotExpr |
| SRF SAP | -0.51 | -5.37 |  | NotExpr |
| Pbx1 | -0.51 | -1.90 |  | NotExpr |
| msx1/2/3 | -0.51 | -7.05 |  | NotExpr |
| HiNF-D3 | -0.51 | -5.48 |  | NotExpr |
| C/EBPalpha | -0.51 | -6.40 |  | NotExpr |
| NFkBp65 | -0.52 | -6.05 |  | NotExpr |
| CBF | -0.52 | -3.69 |  | NotExpr |
| PREB | -0.54 | -5.32 |  | NotExpr |
| HNF1a/b/c | -0.54 | -6.81 |  | NotExpr |
| alpha-PAL | -0.54 | -6.75 |  | NotExpr |
| ABF-1 | -0.54 | -6.13 |  | NotExpr |
| Mfh-1 | -0.56 | -4.72 |  | NotExpr |
| IL-6 RE-BP | -0.56 | -4.85 |  | NotExpr |
| c-Myc | -0.56 | -4.61 |  | NotExpr |
| CBFB | -0.56 | -7.27 |  | NotExpr |
| MHC W box | -0.57 | -0.49 |  | NotExpr |
| ISGF | -0.57 | -0.74 |  | NotExpr |
| c-fos BP | -0.57 | -5.10 |  | NotExpr |
| XBP1 X2BP | -0.59 | -5.02 |  | NotExpr |
| PPUR(1) | -0.59 | -6.40 |  | NotExpr |
| TGT3 | -0.60 | -5.59 |  | NotExpr |
